# Impacts of Temperature and Water Activity Interactions on Growth, Aflatoxin B1 Production and Expression of Major Biosynthetic Genes of AFB1 in *Aspergillus flavus* Isolates

**DOI:** 10.3390/microorganisms11051199

**Published:** 2023-05-04

**Authors:** Mayasar I. Al-Zaban

**Affiliations:** Department of Biology, College of Science, Princess Nourah bint Abdulrahman University, P.O. Box 84428, Riyadh 11671, Saudi Arabia; mialzaban@pnu.edu.sa

**Keywords:** *Aspergillus flavus*, aflatoxin biosynthesis genes, real-time PCR, water activity, temperature

## Abstract

The contamination of peanuts, with *Aspergillus flavus* and subsequent aflatoxins (AFs) is considered to be one of the most serious, safety problems in the world. Water activity (a_w_) and temperature are limiting, factors for fungal growth and aflatoxin production during storage. The objectives of this study were to integrate data on the effects of temperature (34, 37, and 42 °C) and water activity (a_w_; 0.85, 0.90, and 0.95) on growth rate aflatoxin B1 (AFB1) production and up- or-downregulation of the molecular expression of biosynthetic AFB1 genes divided into three types based on their *A. flavus* isolate composition and AFB1 capacity in vitro: *A. flavus* KSU114 (high producer), *A. flavus* KSU114 (low producer), and *A. flavus* KSU121 (non-producer). The *A. flavus* isolates were shown to be resilient in terms of growth on yeast extract sucrose agar media when exposed to temperature and water activity as pivotal environmental factors. The optimal conditions for the fungal growth of three isolates were a temperature of 34 °C and water activity of 0.95 a_w_; there was very slow fungal growth at the highest temperature of 42 °C, with different a_w_ values causing inhibited fungal growth. The AFB1 production for the three isolates followed the same pattern with one exception: *A. flavus* KSU114 failed to produce any AFB1 at 42 °C with different a_w_ values. All tested genes of *A. flavus* were significantly up- or downregulated under three levels of interaction between temperature and a_w_. The late structural genes of the pathway were significantly upregulated at 34 °C under a_w_ 0.95, although *aflR*, *aflS* and most of the early structural genes were upregulated. Compared to 34 °C with an a_w_ value of 0.95, most of the expressed genes were significantly downregulated at 37 and 42 °C with a_w_ values of 0.85 and 0.90. Additionally, two regulatory genes were downregulated under the same conditions. The expression level of *laeA* was also completely associated with AFB1 production, while the expression level of *brlA* was linked to *A. flavus* colonization. This information is required to forecast the actual effects of climate change on *A. flavus*. The findings can be applied to improve specific food technology processes and create prevention strategies to limit the concentrations of potential carcinogenic substances in peanuts and their derivatives.

## 1. Introduction

Peanut (*Arachis hypogaea* L.) is an important economic crop for oil production and a nutritious addition to, human consumption. One of the major problems associated with peanut production worldwide is aflatoxigenic *Aspergillus* isolates, whose contamination and the aflatoxin (AF) production are massive human health risks that can cause huge economic losses for the peanut industry. AFs are polyketide-derived furanocoumarins with powerful carcinogenicity that are linked with both acute and chronic toxicity for humans and animals [1,2]. Aflatoxin B1 (AFB1), the most risky and toxic of the Afs, is typically produced at high levels by select isolates of aflatoxigenic *Aspergillus*. Therefore, controlling AFB1 contamination requires a special focus on investigating the growth and metabolism of *A. flavus*, particularly AFB1 biosynthesis [3]. The 75-Kb gene cluster of *A. flavus* contains genes encoding the AFs biosynthesis pathway. To date, 34 genes have been discovered and identified, and their functions have been recognized as basic genes of the AFs pathway gene cluster [4]. There has been interest in the implications of climate-change-related abiotic conditions such as water activity (a_w_) and temperature, which are expected to affect the post-harvest phase of peanut crop, particularly by increasing the risk of fungal growth, aflatoxin production and the expression of aflatoxin pathway genes [5]. Strong effects of environmental conditions, such as temperature and a_w_ modifications, on the functioning of storage ecosystems and deleterious *A. flavus* and suppression of the AF genes that prevent aflatoxin production lead to AF gene suppression, which represents a key step in risk management [6]. Two factors impact the expression of the AFB1 regulatory system and therefore AFB1 production: first, all biosynthetic genes, enzymes and regulators of AFB1 (an extremely complex molecular system), and second, storage ecosystems such as the a_w_, temperature and CO2 concentration [7]. It was established that the ratio of the two main regulatory genes (*aflS*/*aflR*) is related to temperature and a_w_ interactions. High production of AFB1 is associated with a significant ratio of *aflS* to *aflR* [8]. An analysis of the AFB1 gene expression profiles revealed that 9 genes had their maximum levels of expression at 37 °C under an a_w_ of 0.92, while 16 of the 25 genes had their highest levels at 28 °C under an a_w_ of 0.92. At 42 °C, all genes involved in the biosynthesis pathway for AFB1 were downregulated compared to at 37 °C [6]. The transcription levels of two regulatory genes and 19 structural genes in the AF gene cluster in *A. flavus* at different temperatures showed significantly different expression levels for environmental factors, such as the interaction between the level of a_w_ and temperature [9]. The transcription levels of two regulatory genes and 19 structural genes in the AFB1 gene cluster in *A. flavus* at different temperatures showed significantly different expression levels for environmental factors, such as the interaction of a_w_ and temperature [9]. Previous research has suggested that interactions between these critical abiotic factors may have a significant impact on fungal diseases of fundamental food crops and possibly on the AFB1 contamination of food and feed. In order to control fungal development and subsequent AFB1 formation on peanut crops, greater effort should be made to obtain optimal storage conditions (especially regarding the a_w_ and temperature) [9,10].

This study contributes to gaining a better understanding of the regulatory mechanisms of multiple combinations of a_w_ and temperature on *A. flavus* development, biosynthetic, pathway gene expression, and AFB1 production, and this information is useful for reducing AFB1 contamination during peanut storage.

## 2. Materials and Methods

### 2.1. Fungal Isolates

Three *A. flavus* type isolates (KSU107, KSU114 and KSU121) provided by Dr. M. Mahmoud (Central Lab. of Biotechnology, Plant pathology Res. Ins. Agricultural Research Center, Giza, Egypt) were used in this study. The isolates had previously been used for the molecular detection of *A. flavus* in stored peanut studies [11] with consistent results. They were stored at 4 °C or subcultured on yeast extract sucrose agar (YES; 2% yeast extract, 15% sucrose, 0.05% MgSO_4_ 7H_2_O).

### 2.2. Fungal Isolates and Growth Conditions

Three isolates were grown for five days at 25 °C on YES agar (20 g/L yeast extract, 150 g/L sucrose and 15 g/L agar). After covering the agar plates with sterile 8.5 cellophane sheets (Bristol, UK), a single-point inoculation was performed in the center of each plate using 10^7^ of a spore suspension made up of 1 × 10^7^ spores in 0.5% tween 80 and 0.85 g/L NaCl. By utilizing glycerol/water mixes to change the water availability to 0.85, 0.90 and 0.95 a_w_, as previously stated, the a_w_ of the media was altered [11]. The water activity of the medium was verified by using an Aqualab 3TE instrument (Decagon, Pullman, WA, USA). The plates were inoculated at the temperatures indicated (34, 37 and 42 °C) with replicates (three per treatment). Samples were divided into three parts to carry out (i) a growth assessment, (ii) quantification of AFB1 by HPLC and (iii) determination of the expression of aflatoxin biosynthesis-related genes by real-time PCR (RT-PCR).

### 2.3. Growth Assessment

The plates of *A. flavus* were measured by taking two diametric measurements at right angles to each other at 3, 5, 7 and 9 days.

### 2.4. Quantification of AFB1 by HPLC

The media material was sliced into 4–5 plugs, each weighing about 0.5 g and placed over 9 cm Petri plates. These were weighed after being put into 2 mL Eppendorf tubes. One milliliter of chloroform was added, and AFB1 was extracted by shaking for one hour. After centrifugation, the chloroform was evaporated to dryness, and the biomass was discarded. Aflatoxin-containing dried residues were dissolved in one milliliter of the same liquid mobile phase solution (methanol: acetic acid: water) and kept in opaque vials. AFB1 production was detected and determined using the method presented in [12,13]. The extract was passed through a 0.45 μm micro-filter. A compound analysis was done using HPLC (PerkinElmer^®^ series 200, Walhtam, MA, USA), C18, 100 × 4.6 mm, 3-micron HPLC type. An UV detector was installed in the HPLC, and its wavelength was 365 nm. The mobile phase was composed of acetic acid, methanol and water (20.0/20.0/60.0 *v*/*v*/*v*). At a flow rate of 1 mL/min, the separation took roughly 25 min to complete.

### 2.5. Extraction of RNA, cDNA Synthesis and the RT-PCR Reaction

Following the manufacturer’s instructions, total RNA was extracted from fungal mycelia using the Plant Mini Kit (QIAGEN, Hilden, Germany). DNA-free DNase was used to process the RNA samples. With the help of the Gen Qunta system, the purity and quantities of RNA were determined by measuring the samples’ absorbance at 260 and 280 nm (Amersham Pharmacia Biotech, Amersham, UK). Takara RNA PCR Kit (AMV) ver. 3.0 was used to reverse-transcribe 5 g of total RNA to produce the cDNA (Takara, Shiga, Japan). The primes were synthesized by GeneLink (Orlando, FL, USA). Table 1 contains a list of the primers used in this study. Reactions using real-time PCR were carried out (Applied Biosystems, Foster City, CA, USA). The amplification detector was the SYBR Green Real-time, PCR Master Mix (Applied Biosystems Foster City, California, USA) with a fluorescent tag. As an internal control, the 18S rRNA sequence was utilized. The following components were used to prepare each reaction (20 μL): 100 ng cDNA, (1 μL), SYBR Premix Ex Taq, (10 μL), Nuclease-Free water (8 μL), and 10 mmol/L primers (0.5 L), both forward and reverse. The PCR protocol had 40 cycles of denaturation at 94 °C for 30 s each, 40 annealing cycles at 57 °C for 30 s, and an initial denaturation phase at 94 °C for 10 min. After the annealing and extension stage, continuous fluorescence signals were produced and monitored once per cycle. At the conclusion of each run, a melting curve was created to guarantee product homogeneity. The 2^−ΔΔCt^ method was used to calculate the relative quantification of changes in gene expression [14]. Each gene’s untreated expression was regarded as the control and given a treatment level of 1.0. In each experiment, each sample was tested in triplicate, and all genes were examined. Each experiment was run three times.

### 2.6. Statistical Analysis

In all experimental trials, three replicates of each treatment were used. The standard error of the means (SE), along with the average of each of the three measures, was used to calculate the means. In order to analyses the variance of means with a 95% confidence interval, the Analysis of Variance (ANOVA) was used. Using the same statistical software, the Least Significant Difference (LSD) was used to determine differences between the means, with *p* 0.05 indicating a significant difference.

## 3. Results

### 3.1. Effect of a_w_ × Temperature Interactions on Three Isolates of A. flavus Grown on YES Agar Media

**Figure 1:**
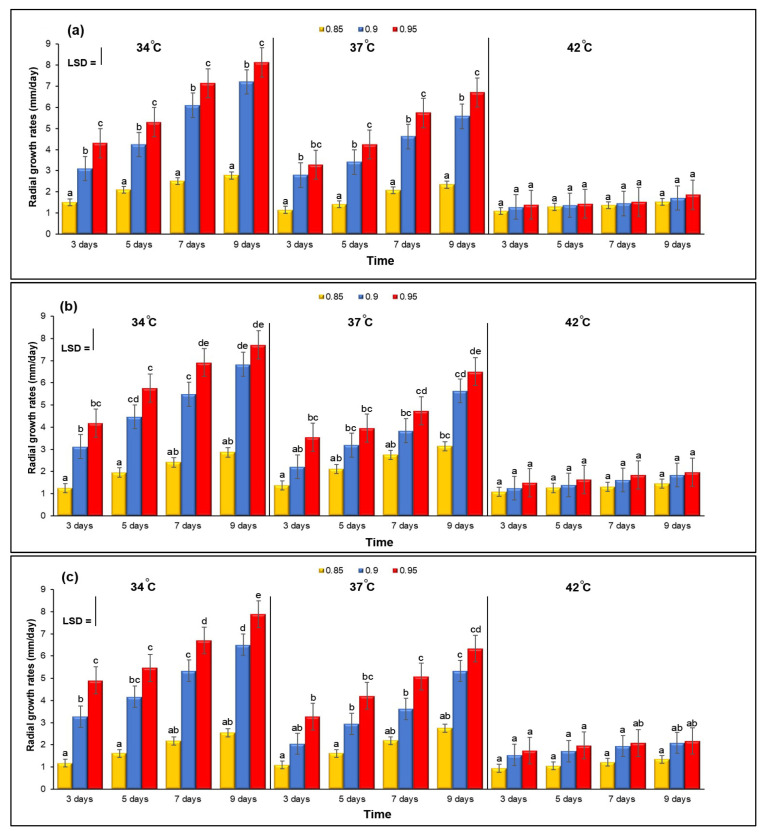
(**a**–**c**) The combined effect of three different levels of a_w_ and temperature on the growth rates. Bars indicate the Least Significant Difference (LSD, *p* ≤ 0.05). Error bars represent the ±Standard Error of the mean of three replicates (*n* = 3). Different letters indicate significant differences.

On YES medium, growth cultures of *A. flavus* KSU107, *A. flavus* KSU114 and *A. flavus* KSU121 was investigated in relation to three different levels of a_w_ and temperature. Figure 1a–c show the combined effect of three different levels of a_w_ and temperature on the growth rate. The growth of the three *Aspergillus* isolates generally increased gradually, especially at the medium temperatures of 34 and 37 °C compared with the high temperature of 42 °C. This shows that the optimal colonization conditions for three isolates were 34 °C and 0.95 a_w_ after nine days; there was very slow fungal growth at the highest temperature of 42 °C, and all a_w_ treatments were inhibited.

### 3.2. Effect of a_w_ × Temperature Interactions on AB1 Production by Three A. flavus Isolates on YES Agar Media

**Figure 2:**
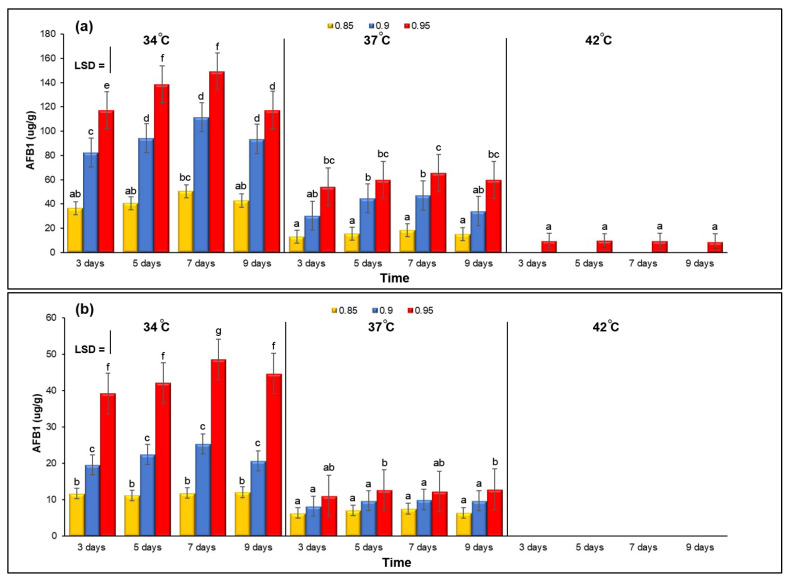
The combined effect of three different levels of a_w_ and temperature on AFB1 production by *A. flavus* KSU107 (**a**), *A. flavus* KSU114 (**b**) grown on YES agar media and incubated at 34, 37 and 42 °C under 0.85, 0.90 and –0.95 a_w_. Error bars represent the ±Standard Error of the mean of three replicates (n = 3). Different letters indicate significant differences.

Figure 2a,b show the combined effect of three different levels of a_w_ and temperature on the concentration of AFB1 in two isolates of *A. flavus* (*A. flavus* KSU107 and *A. flavus* KSU114) examined on YES agar media. Generally, AFB1 production was higher at 34 °C with 0.95 a_w_ for both isolates. When comparing samples grown at 34 °C to those grown at other temperatures, AFB1 production was significantly increased at 34 °C and 0.95 a_w_. At 42 °C with 0.85 and 0.90 a_w_, both isolates failed to produce any amount of AFB1. *A. flavus* KSU121 failed to produce any AFB1 at different temperatures and a_w_ levels.

### 3.3. Effect of a_w_ × Temperature Interactions on the Expression of 15 Genes Involved in the Biosynthesis of AFB1 Production

**Figure 3:**
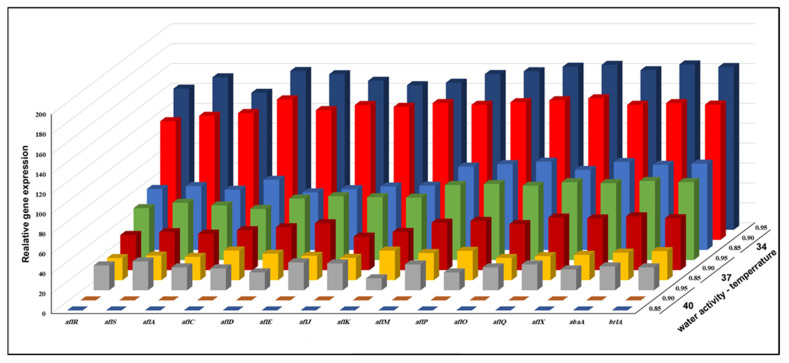
The combined effect of three different levels of a_w_ and temperature on the relative expression of fifteen AFB1 genes of *A. flavus* KSU107 (high AFB1 producer) grown on a YES agar medium.

**Figure 4:**
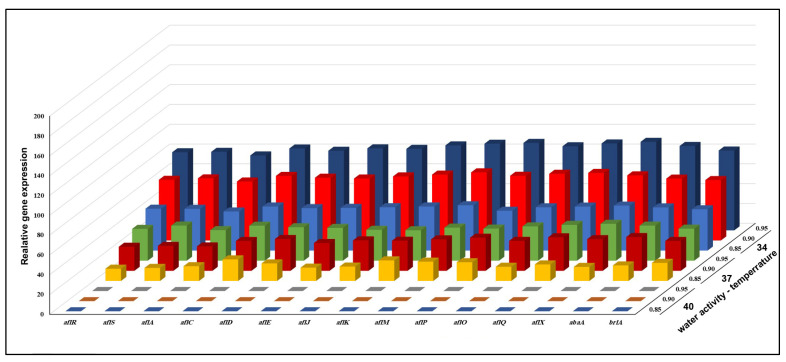
The combined effect of three different levels of a_w_ and temperature on the relative expression of fifteen AFB1 genes of *A. flavus* KSU114 (low AFB1 producer) grown on a YES agar medium.

The RT-PCR investigation of *A. flavus* KSU107 (high AFB1 producer) and *A. flavus* KSU114 (low AFB1 producer) revealed the transcription levels of 15 genes implicated in the production of AFB1 with combinations of three levels of a_w_ and temperatures after 7 days of cultivation. Figure 3 and Figure 4 show the combined effect of three different levels of a_w_ and temperature on the transcription levels of 11 structural genes in the AFB1 gene cluster, two regulatory genes (*aflR* and *aflS*) and two fungal development regulators (*abaA* and *brlA*) in both *A. flavus* isolates at different a_w_ × temperature combinations to indicate the molecular mechanism underlying the up-and-downregulation of the molecular expression profile of the tested AFB1 biosynthetic genes. Figure 3 indicates the transcription levels of previously mentioned regulatory genes in *A. flavus* KSU107 (high AFB1 producer) at various a_w_ levels and temperatures. In general, the majority of the examined genes had significantly different expression levels under various a_w_ x temperature combinations. All of the AFB1 biosynthesis-related genes were evaluated and showed the highest levels of expression at 34 °C with 0.95 a_w_ compared to at 37 °C and 42 °C with various a_w_ values. The five most upregulated genes were *aflO*, *aflQ*, *aflX*, *abaA*, and *brlA*; these genes were upregulated by an average of 2.1-fold at the same temperature and 0.80 a_w_. At 37 °C with 0.95 a_w_, all of the tested genes were linked to AFB1 production. When converting Versicolorin A (VERA) to AFB1, the middle and late genes involved in the -AFB1 biosynthetic pathway were significantly upregulated, with a 1.25- to 5.5-fold increase when compared to conditions of 37 °C and 0.85 a_w_. At 40 °C with 0.85 and 0.90 a_w_, none of the genes in the biosynthetic pathway, which involved regulatory and structural genes, were expressed. 

Changes in the gene expression of all genes by KSU114 (a low AFB1 producer) after a 7-day incubation period based on interactions between different temperatures and a_w_ values are shown in Figure 4. Overall, combinations of three levels of a_w_ and temperature had a substantial impact on the majority of the detected genes. The highest levels of gene expression were observed at 34 °C and 0.95 a_w_, while at 40 °C, with all a_w_ levels, nonexpression was observed for all genes. At 37 °C with 0.95 a_w_, tall AFB1 biosynthetic pathway genes were highly upregulated with 3.25- and 2.50-fold increases compared with the levels at 37 °C with an a_w_ of 0.95 and 78.8- and 80.5-fold increases compared with the levels at 42 °C with an a_w of_ 0.95, respectively. The four AFB1 genes with the highest levels of expression were *aflM*, *aflP, aflQ* and *aflX* at 34 °C with an a_w of_ 0.95. Additionally, at 37 °C with an a_w_ of 0.95, the same effect was observed for the *aflK*, *aflM*, *aflO* and *aflQ* genes.

## 4. Discussion

According to the literature, *A. flavus* can develop and produce AFB1 across a wide range of a_w_ values and temperatures [6]. Numerous studies have described the ideal a_w_ temperature for *A. flavus* growth and AFB1 production on various food substrates and formula media [15,16,17]. *A. flavus* growth and AFB1 production on polished rice can occur over a broad range of a_w_ × temperature levels. For fungal growth, the optimal conditions were found to be an a_w_ of 0.92–0.96 and a temperature of 28–37 °C. The maximum quantity of AFB1 was observed at 33 °C and an a_w_ of 0.96 [18,19]. The A. flavus isolates were elastic for the duration of their growth on pistachio-based media and the colonization of pistachio nuts with no significant difference when exposed to temperature (35 vs. 37 °C) and water activity (0.98–0.93) as abiotic factors related to climate change (CC). AFB1 production was significantly promoted, particularly when exposed to a temperature of 35 °C and an a_w_ of 0.98–0.95 and occasionally at 37 °C and 0.98 a_w_ [20]. The analysis of variance for the effects of temperature and a_w_ on the radial growth of two isolates of *A. flavus* revealed that these variables as well as their interactions had substantial impacts on fungal growth and the formation of AFB1. The response surface analysis revealed that both isolates could develop at temperatures between 22 and 37 °C and a_w_ values between 0.88 and 0.98, although the production of AFB1 did not occur at temperatures below 37 °C at 0.90 a_w_. The authors suggest that the a_w_ in harvested chili should be immediately decreased to less than 0.90 by an effective drying method to prevent fungal growth and AFB1 production [21]. Two *A. flavus* isolates isolated from imported raw peanuts were studied for growth and AFB1 production using a full factorial design with seven a_w_ levels (0.85–0.98 a_w_) and five temperature levels (20–40 °C). The findings showed that both isolates were unable to grow at 20 °C under a_w_ values of 0.94 and 0.85. At 30 °C with an a_w of_ 0.98, both isolates showed the maximum growth rate. According to the analysis of variance (*p* < 0.05), temperature and a_w_ had significant impacts on *A. flavus* growth and AFB1 production. AFB1 production showed a similar pattern, but at a lower temperature range (25–35 °C) and higher a_w_ (0.92–0.98) [22]. *A. flavus* grew on shelled peanuts at a lower rate when the water activity was ≤0.85 or the temperature was ≤20 °C. A temperature of 37 °C and a_w_ of 0.98 were the best conditions for fungal growth in shelled peanuts. The maximum amount of AFB1 in peanuts was obtained at a_w_ of 0.96 and 28 °C [6]. Temperature and a_w_ represent two key environmental factors that influence both the rate of fungal colonization of food and feed and the production of AFs. Previous studies have shown that a_w_ and temperature significantly affect the expression of genes implicated in the production of AFB1, particularly the regulatory genes *aflR* and aflS [12]. Gene expression continuously precedes the appearance of phenotypic traits. Genome sequencing and many annotations of *A. flavus* have paved the way for various molecular approaches, such as reverse transcription real-time PCR (RT-qPCR) and RT-PCR, which may be useful to obtain crucial information on the up-and downregulation of specific genes involved in AFB1 biosynthesis under various environmental conditions [23]. RNA-Seq technology was used to determine the effect of temperature on aflatoxin biosynthesis, and it was discovered that most genes involved in the aflatoxin cluster were highly expressed at 30 °C but not at 37 °C, because the very low concentration of salts in the medium which supported *A. flavus* did not produce a high concentration of AFs [24]. In comparison to 37 °C, [25] found that a medium temperature (30 °C) upregulated the expression of the genes *aflR* and *aflS*, activating the AFB1 biosynthesis gene. The transcriptional activation and inactivation of the AFB1 gene cluster appear to be modulated by slight variations in the expression levels of *aflR* and *aflS*. Additionally, [6] found a similar result for AFB1 production in peanut kernels. At 28 °C, a higher ratio of *aflS*/*aflR* led to a higher level of AFB1 in peanut kernels compared with 42 °C and 37 °C. According to qRT-PCR results, the two regulating genes (*aflR* and *aflS*) were significantly expressed at varied conditions such as (20 and 28 °C and 0.96–0.99 a_w_) during AFB1 production. Additionally, under the conditions required to achieve the highest capacity for AFB1 production, two structural genes (*aflD* and *aflO*) were significantly expressed [26]. At various a_w_ and temperatures, the majority of the examined AFB1 production genes displayed noticeably differential expression levels. All of the AFB1 biosynthetic development-related genes evaluated showed the highest transcription levels at 25 °C under an a_w_ of 0.96, compared to at 33 °C and 37 °C. The two most highly expressed genes were *aflR* and *aflS* (regulatory genes), followed by *aflE*, *aflJ*, and *aflH* (structural genes) [19]. In the study, the relative transcription levels of *abaA* and *brlA* were significantly upregulated at 37 °C compared to at 40 °C, and they were highly significantly upregulated at 34 °C. In comparison with at a_w_ 0.95, the transcription levels of both genes were also significantly reduced at a_w_ values of 0.85 and 0.90 under different temperatures. Similar findings were observed by [6]. When *A. flavus* grew on peanut kernels at 33 °C under different a_w_ conditions, a sufficiently favorable correlation between these conditions and the expression levels of the *abaA* and *brlA* genes was observed [6], whereas a similar correlation was not found for polished rice [19]. To trigger the transcription of each of the structural genes in the AFB1 pathway in the AFB1 gene cluster, *aflS* and *aflR* join forces to form a complex known as the AflR binding molecule, which then binds to the promoter region of each structural gene and activates it. Upregulation of *aflS* resulted in high AflS production, which could subsequently provide a good opportunity for the formation of the AflR-AflS complex, causing increases in AFB1 gene transcription and AFB1 production [27]. The *aflR* and *aflP* genes experienced substantial peaks in expression for both *A. flavus* and *A. parasiticus* species in maize kernels by day 6 of incubation, while the *aflS* gene reached its peak by day 8. Despite the fact that the relative gene expression levels were relatively similar, the *A. parasiticus aflP* gene expression values in this food matrix were greater than those for *A. flavus*. In peanut seeds, greatest expression values for the *aflR*, *aflS* and *aflP* genes were seen at 7, 9 and 8 days of incubation for *A. flavus*. However, the three AF-related genes’ greatest expression levels were observed in *A. parasiticus* by day 7 of incubation. *A. parasiticus* obtained greater relative expression values for the *aflP* gene than did *A. flavus*. *A. flavus* was discovered to have the highest expression levels of the *aflS* gene [28]. Senna pods are very sensitive to *A. flavus* and AFB1 contamination, and the regulatory restrictions of importing countries make it difficult for pods to be successfully exported. The diametric growth rate was shown to be most optimal at 28 and 37 °C with an a_w_ of 0.96. However, regulatory gene expression (*aflR* and *aflS*) was highly expressed and AFB1 production was at its highest at 28 °C with an a_w_ of 0.96. The maximum growth rate was seen at 37 °C, which was favorable for neither gene expression nor the formation of AFB1. However, at 28 °C, it was positively correlated with gene expression and AFB1 production. The difference in the growth rate and AFB1 production at the highest temperature may have been caused by the suppressed molecules’ effect on the expression of regulatory genes. The rapid drying of senna, pods with a low a_w_ (≤0.87 a_w_) and storage at a low temperature (20 °C) are ideal conditions for avoiding AFB1 and ensuring the quality of produce for export [29]. The *A. flavus* isolate NRRL3357 was inoculated in peanuts with diverse a_w_ values (0.90, 0.95 and 0.99). The changes in AFB1 production were the highest with a_w_ 0.90, followed by a_w_ 0.95 and were minimal at a_w_ 0.99. According to the transcriptome data and RT-qPCR analyses, the middle- and later-stage genes involved in AFB1 biosynthesis in particular were considerably upregulated in a_w_ 0.90 compared to at a_w_ 0.95 and 0.99. The AtfB factor may play a key role in regulating downstream genes, especially AFB1 biosynthetic genes, in response to a_w_ changes [5]. The stress of the environmental factors associated with climate change, such as drought, temperature and oxidative stress, can aggravate aflatoxin contamination in agricultural crops [30]. Over the last decade, abundant research efforts have been undertaken to confirm that environmental stress affects aflatoxigenic *A. flavus* and AF production. Environmental stress-responding compounds, such as reactive oxygen species (ROS), which are generated by host cells in oxidative bursts associated with fungal stress-responsive signaling and secondary metabolites, can stimulate the production of AFs by *A. flavus*. Both temperature and aw changes and oxidative stress are tightly linked to ROS metabolism in fungal cells. Given that ROS play important roles in the regulation of both fungal metabolites and AF production, a serious analysis of the responses of *A. flavus* to all stresses and the regulation of AFs production is supported, thereby aiding efforts to prevent AF contamination under climate change scenarios [31,32]. Indeed, fungal cells have a complex “temporal switch” that regulates the activation of AF genes involved in AFs biosynthetic pathways and controls their activity. The transcriptomic responses of *A. flavus* to temperature, a_w_ and oxidative stresses at the AF production stage revealed that AF biosynthetic genes were strongly affected, which means that AF genes were upregulated under stress conditions [33]. Advanced genomic techniques have greatly improved our knowledge of the molecular aspects of the regulation of AF biosynthesis genes. However, data on AF regulation at the molecular level under climate change scenarios are still very scarce. The availability of up-to-date techniques, including genomics and transcriptomics, has contributed to a prompt expansion of data on the biology of *A. flavus* as a mycotoxigenic fungus [34]. The availability of up-to-date techniques, including genomics and transcriptomics, has contributed to a prompt expansion of data on the biology of *A. flavus* as a mycotoxigenic fungus. Indeed, the use of transcriptomic data is a better way to understand AF modelling, climate change and development. Additionally, transcriptomics explains how chromatin remodeling is influenced by interacting stresses of environmental factors and a more comprehensive view of climate change scenarios. Transcriptional regulation of AF production is dependent on diverse factors, including AF cluster genes, regulators, transcription factors, environmental factors, and crop substrates. Transcriptomic and proteomic analyses produce accurate data that can be used to identify the genes or pathways involved in the regulation of AF biosynthesis [35,36].

## 5. Conclusions

The current study examined the impacts of temperature and a_w_ on the development of three isolates of *A. flavus*, the transcription of important regulatory and structural genes and the biosynthesis of AFB1 on YES agar media. The findings showed that a_w_ and temperature have significant effects on the expression of the critical genes involved in AFB1 biosynthesis in all isolates of *A. flavus* as well as having effects on AFB1 production and fungal growth. The expression of structural genes is more strongly influenced by a_w_ and temperature than by AF’s regulatory genes, *aflR* and *aflS*. The findings presented here may provide readers with a deeper understanding of the decrease in AFB1 production caused by peanut contamination. The two main environmental parameters that affect the speed of fungal growth and AFB1 formation in the storage and marketing processes are a_w_ and temperature. AFB1 production and fungal spoiling of peanuts and peanut products may be prevented with the help of the information that is provided here regarding crucial control.

## Figures and Tables

**Table 1:** Primers used for real-time PCR.

Genes	Length (bp)	Primer Sequence
*aflR*	88	F: CCTTTCTCACTACTCGGGTTT R: GCAGGTAATCAATAATGTCGG
*aflS*	109	F: CTCGATGCGGCAGTGTATCT R: ACACCTCCACATGAGCCTTG
*aflA*	111	F: CATGCTGTTAACCCCCGACT R: AATTGGGCTAGGAAACCGGG
*aflC*	61	F: TGCATGGCGATGTGGTAGTT R: GTAAGGCCGCGGAAGAAAG
*aflD*	106	F: ATGCTCCCGTCCTACTGTTT R: ATGTTGGTGATGGTGCTGAT
*aflE*	134	F: GTGTGGAGGAAGTGATGCGA R: CGGGGTAAGTCCGTTAGCTC
*aflJ*	146	F: GCGTGATCAGTCGTCAATGC R: CAGGATGAGCGGTTGGTTCT
*aflK*	114	F: GCTGGGCATTCCAGTACGAT R: CCCATCAACTGACTGTGGCT
*aflM*	100	F: GAGCCAAAGTCGTGGTGAAC R: GCCTGGATTGCGATAGCGTC
*aflP*	109	F: CACGCTTTCAGAGCAGGTAA R: TTCGGTGGAGGAGGGAGTT
*aflO*	115	F: GTCCCGTTTCCTGGGTTGAT R: GCTTTCGATTGCTGCCCAAA
*aflQ*	134	F: GCACCAACAATTCGGCTCTG R: TGTGGAAGGGTGGAAGATGC
*aflX*	112	F: AGTCCTCAACATAGCCGCTG R: TAGTCCCCCAGGTTTGACGA
*abaA*	120	F: ACTGGCAAAAGGAGGTCGAG R: ATTCGAACGGTCTGCTGGTT
*brlA*	131	F: TCTAGCGGGGATGACCTCAA R: CCGAAGGAAGCCAAAAGTGC

## Data Availability

The data presented in this study are available on request from the corresponding author.
